# Comparative Evaluation of Regeneration Strategies for Rhodamine B Removal Using Raw Date Seeds: Insights into Adsorption Mechanisms

**DOI:** 10.1021/acsomega.6c01845

**Published:** 2026-06-11

**Authors:** Tugba Celik Caglar, Birol Kayranli

**Affiliations:** Department of Environmental Science, Gazi University, Ankara 06560, Turkey

## Abstract

Rapid industrial development has led to a substantial increase in the discharge of dyestuffs into the environment, leading to severe environmental contamination. Elevated concentrations of dyes in aquatic systems have raised significant concerns due to their toxicity, persistence, and adverse ecological effects, thereby motivating extensive research into effective wastewater treatment strategies. Among the various remediation techniques, adsorption has gained considerable attention as an efficient, cost-effective, reusable adsorbent and environmentally friendly method for dye removal from aqueous media. This study investigates the adsorption mechanism of Rhodamine B (RhB) onto raw date seed as a low-cost biosorbent, with particular emphasis on surface interactions, kinetic behavior, and regeneration performance. Equilibrium isotherm analysis revealed that RhB adsorption proceeds on a heterogeneous surface via a predominantly multilayer mechanism, with the Freundlich and Dubinin–Radushkevich models providing the most accurate representation of the experimental data. Kinetic results showed excellent agreement with the pseudo-second-order model, indicating that surface interactions govern the rate-limiting step. Zeta potential analysis showed that the adsorbent surface becomes increasingly negatively charged, indicating that electrostatic interactions are a major contributing factor in the adsorption process. Accordingly, maximum RhB removal efficiency was achieved under pH:3, 1.0-g adsorbent, 303.0 K, and with 30.0 min contact time. Regeneration, SEM, and FTIR analyses revealed that adsorption–desorption performance strongly depends on the regeneration strategy, with the fourth regeneration method, which combined NaOH treatment with high-temperature drying at 120 °C, most effectively preserving surface morphology and pore accessibility. Furthermore, the RhB removal efficiencies obtained using the fourth regeneration method were 96.3 and 88.1% in the first and fourth cycles, respectively. Overall, the findings establish raw date seed as an efficient, regenerable, and mechanistically well-defined biosorbent for RhB removal from aqueous systems.

## Graphical abstract

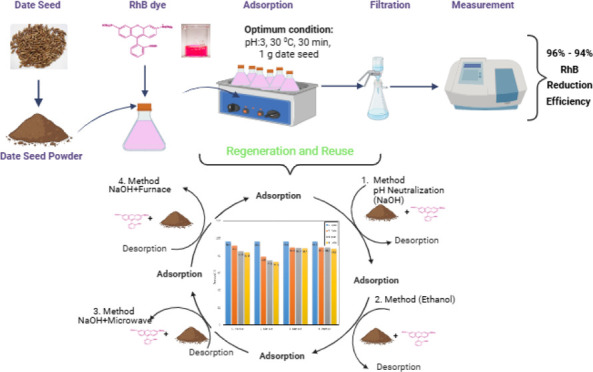

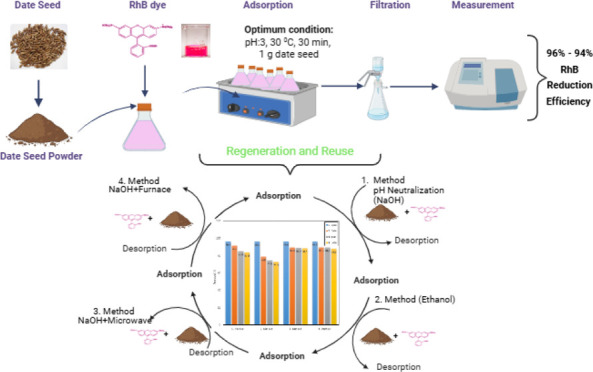

## 1 Introduction

Industrial production of textiles is a long and complex process in which natural or synthetic fibers are transformed into yarn and fabric. The increasing amount of hazardous dye wastewater generated by various industries continues to pose a serious public health concern and a major environmental problem, presenting significant challenges for existing conventional wastewater treatment plants. From the initial sizing to the final washing steps, numerous chemical-based processes cause severe environmental issues in textile industries.^1^ In terms of wastewater generation, the study conducted by Shaikh indicated that the production of 9 tons of textile products requires 36 tons of water, ranking second among industries in terms of wastewater generation.^2^ Approximately 54% of wastewater is produced during bleaching and dyeing processes, in which a wide variety of dyes and chemicals are used.^2^

Rhodamine B is a dye widely used in textile dyeing, the food industry, and biomedical laboratories as a colorant.^2^ Rhodamine B is a basic red, water-soluble, and typically fluorescence-tracing dye with photophysical properties. In aqueous systems, Rhodamine B (RhB) may occur in three structural forms: zwitterionic, cationic, and lactonic. The cationic form predominates under acidic conditions, whereas the zwitterionic species becomes dominant in alkaline media. Conversely, the lactonic form is favored only in nonpolar or polar aprotic solvents.^3^ Belonging to the xanthene class of dyes, it is one of the oldest synthetic dyes used in the textile and food industries. The presence of N-ethyl groups on both sides of the xanthene rings renders it toxic and carcinogenic to humans and animals, and therefore, its use in food and cosmetic products has been banned.^4^ Rhodamine B is not only harmful when ingested but also causes severe acute oral toxicity. It can lead to serious damage and irritation in the eyes, as well as long-term hazardous effects on aquatic environments.^5^ Particularly in textile industry effluents, significant concentrations of Rhodamine B have been detected, which continues to be both a major public health and environmental issue while posing a serious challenge to existing conventional water treatment systems. Due to its significant environmental impact, the discharge of untreated dye-containing wastewater is subject to strict regulations, requiring compliance with limits on parameters such as color and COD before discharge. Consequently, physicochemical and biological treatment processes have been investigated for wastewater remediation.^6^ The direct discharge of untreated dye-containing wastewater into the environment has detrimental effects on the photosynthetic activity of aquatic ecosystems. Due to the presence of metals and aromatics, it also exerts mutagenic or teratogenic effects on aquatic organisms and fish species.

Various wastewater treatment methods exist, including adsorption, membrane filtration, and advanced oxidation processes, with adsorption being among the most effective and practical approaches.^7^ Several novel technologies have been applied for dye removal from wastewater, such as photocatalytic degradation,^8^ ultrasonic degradation,^9,10^ sonochemical degradation,^11^ oxidative degradation,^12^ electrochemical oxidation,^13^ ion flotation,^14^ liquid–liquid extraction via membrane technology,^15^ flocculation, quantum dots (QDs)^16^, and adsorption using various inorganic and organic-based materials;^17,18^ . Among these methods, adsorption using biomass-derived materials has been found superior due to its lower initial cost, eco-friendly nature, simple and flexible design, ease of operation, and low maintenance requirements.^18^ Food residues, commonly found in many countries, have demonstrated similar adsorbent capacities to activated carbon. Researchers are showing growing interest in using carbon-rich materials for dye removal due to their beneficial properties, such as porous structures, large surface areas, and strong catalytic activity^19^

Various lignocellulosic agricultural wastes have been extensively explored as low-cost biosorbents for dye removal from aqueous systems. Biomasses such as rice husk, coconut shell, banana peel, and water hyacinth contain abundant functional groups (e.g., hydroxyl, carboxyl, and phenolic), which facilitate adsorption through electrostatic interactions and surface complexation, with removal efficiencies depending on biomass type and treatment conditions.^20,21^ In addition, lignocellulosic residues such as sugar cane bagasse and corn cob have exhibited higher adsorption capacities exceeding 160 mg g^–1^, highlighting the strong potential of biomass-derived adsorbents as sustainable and cost-effective alternatives to conventional materials.^22–24^ Recent studies have further demonstrated that biomass-derived activated carbons obtained from agricultural and forestry wastes exhibit superior adsorption performance due to their high surface area and well-developed porous structures. However, these activation processes often require additional chemical reagents and energy input, increasing cost and environmental impact^25–27^

Among these materials, date seeds have attracted considerable attention due to their high carbon content and lignocellulosic structure, and have been successfully applied in dye adsorption and heavy metal removal, with reported adsorption capacities of around 38 mg g^–1^ for organic dyes.^28^ Furthermore, modified date seed-based materials such as activated carbons and magnetic composites have demonstrated enhanced adsorption performance.^29^ However, most previous studies have primarily focused on adsorption capacity or material modification, while systematic investigations on regeneration strategies remain limited, highlighting the need for further studies to evaluate the regeneration performance and long-term applicability of date seed-based adsorbents.

In recent years, increasing attention has been directed toward the utilization of date seeds, which are rich in carbon and possess a lignocellulosic structure, for the production of cost-effective and environmentally friendly adsorbents. The date palm is cultivated in regions of Middle-South Asia and North Africa. A date tree begins to bear fruit within 5–8 years and can continue to produce fruit for up to 100 years.^18^ Approximately 7.5 million tons of dates are consumed annually worldwide.^30^ The seed represents 10–18% of the fruit’s weight, resulting in nearly 750,000 tons of date seed waste annually. Date seed waste has traditionally been used as animal feed in cattle, sheep, camel, and poultry industries. Moreover, due to its rich chemical composition, date seeds have been explored for use in food products, such as extracting date seed oil for bakery applications as a dietary fiber source, or roasting seeds to produce a caffeine-free coffee-like beverage.^31^ Chemically, date seeds contain 55–65% carbohydrates (40–57% cellulose, 20–35% hemicellulose, and 15–35% lignin), 10–20% crude fiber, 7–10% fat, 5–7% protein, 5–10% moisture, and 1–2% ash.^32^ This composition makes them suitable candidates for conversion into adsorbents. Currently, in waste management, date seeds are considered food waste and regularly disposed of in landfills, which causes negative environmental impacts. Therefore, designing an environmentally sustainable alternative to convert such waste into a valuable resource is highly beneficial. Consequently, converting date seed waste into adsorbents represents one of the most promising and advantageous valorisation methods, while also providing economic benefits to the date processing industry.^18^

The regeneration of exhaust adsorbents is essential for ensuring both economic and environmental sustainability in adsorption-based wastewater treatment.^33^ Chemical regeneration employs acidic, alkaline, or solvent-based eluents to desorb pollutants, offering simplicity but sometimes causing structural deterioration after repeated use.^34^ Rhodamine B adsorption is most favorable under acidic conditions (around pH ≈ 3), considering previous research, whereas the adsorption efficiency decreases significantly when the pH exceeds pH 5 due to changes in surface charge and dye speciation. Therefore, the solution pH was adjusted to approximately neutral conditions (pH ≈ 7) to promote the desorption process. In particular, NaOH was chosen due to its low cost, wide availability, and frequent use in adsorption–desorption regeneration studies. The alkaline environment created by NaOH weakens the interaction between the adsorbent surface and the dye molecules by altering the surface charge and promoting electrostatic repulsion, thereby facilitating the desorption of Rhodamine B. Ethanol was selected as a representative organic solvent because it can effectively dissolve and desorb organic dye molecules while minimizing structural damage to the biosorbent. Thermal regeneration, widely applied in industry, removes adsorbed organics through controlled heating, though it requires high energy and may alter the porous structure of the adsorbent.^35–37^ Recently, microwave-assisted regeneration has emerged as a rapid and energy-efficient alternative, providing uniform heating and reduced structural damage, while maintaining adsorption efficiency over multiple cycles.^38^ Thus, selecting an appropriate regeneration method depends on adsorbent type, pollutant characteristics, and operational cost considerations.

However, to the best of our knowledge, the use of raw date seed for the reduction of dye substances has not been explored extensively. There is a limited study on RhB removal using date seed. Additionally, it is the first time date seed was regenerated and reused by using different methods as the adsorbent in this study. This study aimed to remove RhB dye by utilizing date seeds as a low-cost adsorbent; to optimize adsorption parameters to establish the maximum adsorption capacity through isotherm and kinetic modeling. Furthermore, chemical, thermal, and microwave regeneration methods were also applied both individually and in combination, and then these results were compared. Finally, to conduct dye removal experiments with regenerated adsorbents to evaluate the efficiency of Rhodamine B removal.

Despite the extensive use of agricultural wastes as biosorbents for dye removal, most previous studies have primarily focused on activated or chemically modified materials to enhance adsorption performance. In contrast, the present study intentionally employs raw date seed without any activation or modification in order to maintain a low-cost and environmentally sustainable approach. The key novelty of this work lies in the systematic and comparative evaluation of multiple regeneration strategies, including chemical, thermal, and microwave methods, for the reuse of raw date seed. To the best of our knowledge, such a comprehensive regeneration analysis for unmodified date seed has not been extensively reported in the literature. Therefore, this study provides new insights into the practical applicability, reusability, and sustainability of raw biomass-based adsorbents, bridging the gap between laboratory-scale adsorption studies and real-world wastewater treatment applications.

## 2 Materials and Methods

### 2.1 Analytical Method

Seeds of Medina date (*Phoenix dactylifera* L.) were obtained from a local supplier, ground into powder, and sieved to obtain a particle size below 250 μm. The powdered seeds were washed with deionized water and subsequently dried in an oven at 343.0 K (Memmert Universal UN750). Rhodamine B (C.I. 45170, Basic Violet 10) was used as the model dye in the adsorption experiments. Analytical-grade reagents, including hydrochloric acid (HCl) and sodium hydroxide (NaOH), ethanol (C_2_H_6_O), were purchased from Merck Chemicals, Turkey, and were used without further purification. All experimental procedures utilized deionized water with a resistivity of 18.2 MΩ·cm, produced by a Merck KGaA Millipore Sigma system (Darmstadt, Germany).

To achieve the target dye concentration, 2.09 g of Rhodamine B (RhB) was dissolved in 1.0 L of deionized water to prepare a stock solution (2,090 mg L^–1^), which was then diluted to the required concentrations. The solution pH was adjusted using 0.1 M HCl or NaOH. Residual RhB was measured at the specified contact times using a UV–Vis spectrophotometer (Lovibond). The properties of the date seed (DS) were characterized as follows: functional groups were identified by ATR–FTIR (Shimadzu IR Spirit-X) at 4 cm^–1^ resolution over 4000–400 cm^–1^; surface area was determined using the Brunauer–Emmett–Teller (BET) method with N_2_ adsorption–desorption in the relative pressure range 0.05–0.30; and the zeta potential of the date seed surface was measured with a Malvern Zetasizer Nano ZSP. The surface morphology of the raw date seed adsorbent before and after adsorption–desorption cycles was examined using scanning electron microscopy (SEM, HITACHI SU8700)

### 2.2 Batch Sorption Experiments

Batch adsorption experiments were carried out in a SELECTA orbital shaking water bath using 50 mL Erlenmeyer flasks, with each experiment performed in triplicate. In each run, 50 mL of Rhodamine B solution of known concentration was mixed with a predetermined amount of adsorbent. The suspensions were agitated at a constant shaking speed of 60 rpm. The initial experimental conditions were set at a Rhodamine B concentration of 0.5 mg L^–1^, adsorbent dosage of 1 g, temperature of 303 K, and shaking speed of 60 rpm in order to determine the optimum pH. During this step, all other parameters were kept constant. Subsequently, adsorption parameters including adsorbent dosage, contact time, temperature, and initial dye concentration were systematically varied, while the remaining experimental conditions were maintained constant to evaluate the individual effect of each variable. All experiments were carried out under batch conditions, and each experiment was performed in triplicate to ensure reproducibility. The absorption spectrum of Rhodamine B was recorded in the wavelength range of 400–680 nm, and the maximum absorbance wavelength (λ_max_) was identified as 553 nm. After adsorption, the suspensions were filtered, and the residual Rhodamine B concentration was determined using a UV–Vis spectrophotometer at this wavelength. According to the UV–vis spectrophotometer calibration curve, *R* ^2^ and equation are 0.9988 and *y* = 0.1969*x*. All experiments were conducted under identical conditions unless otherwise stated. The detailed experimental conditions are summarized in Table 1.

**Table 1:** Experimental Conditions for Batch Adsorption Studies

parameter	range/value
pH	2, 3, 4, 5, 6, 7, 8
adsorbent dosage (g)	0.5, 1.0, 1.25, 1.5, 1.75
contact time (min)	2, 4, 6, 8, 10, 20, 30, 40, 50, 60, 70
temperature (°C)	30, 45, 60
initial dye concentration (mg L^–1^)	0.5–5
shaking speed (rpm)	60
measurement wavelength (nm)	553

Residual dye concentration was determined by withdrawing 2.5 mL samples, filtering, and measuring absorbance at 553.0 nm using a UV spectrophotometer. The adsorption capacity (*q*_e_) and percentage removal (*R*) were calculated using eqs 1 and 2.

$$q_{e}=\frac{C_{0}-C_{e}}{m}\times V$$ 1

$$R=\frac{C_{0}-C_{e}}{C_{0}}\times 100$$ 2

where *q*_e_ (mg g^–1^) represents the amount of dye adsorbed per unit mass of adsorbent, *C*_0_ (mg L^–1^) is the initial dye concentration, *C*_e_ (mg L^–1^) is the equilibrium dye concentration, *V* (mL) is the solution volume, *m* (g) is the adsorbent mass, and *R* (%) denotes the dye removal efficiency.

### 2.3 Kinetic Modeling

The adsorption kinetics of Rhodamine B (RhB) were analyzed using pseudo-first-order, pseudo-second-order, Elovich, and intraparticle diffusion models to better understand the adsorption mechanism. The equations of these models are presented in Table 2. The kinetic parameters were obtained from the linear plots of the respective models, and the goodness of fit was evaluated using the correlation coefficient (*R* ^2^).

**Table 2:** Adsorption Mechanism of RhB onto Date Seed, Equilibrium, and Kinetic Data

isotherms	equations	parameter	298 K	313 K	333 K
Freundlich	log*q*_e_ = log*K*_F_ + $\frac{1}{n}$ log*C*_e_	*K*_F_	10.64	8.76	10.22
		1/*n*	0.66	0.35	0.58
		*R* ^2^	0.99	0.94	0.93
		MPSD	13.95	29.76	33.80
		NSD	10.83	24.30	26.32
		ARE	8.53	19.98	21.44
Langmuir*	$\frac{C_{e}}{q_{e}}=\frac{1}{q_{\max}K_{L}}+\frac{C_{e}}{q_{\max}}$	*q*_m_	1.1027	1.8149	4.2265
	$R_{L}=\frac{1}{1+C_{i}K_{L}}$	*K*_L_	0.3656	0.1356	0.3081
		*R*_L_	0.4876	0.0687	0.0314
		*R* ^2^	0.9979	0.9312	0.9874
		MPSD	1186.9	1123.1	1133.1
		NSD	897.5	917.0	925.2
		ARE	765.5	772.8	774.9
Temkin	*q*_*e*_ = *q*_m_ ln *K*_t_ + *q*_m_ ln *C*_e_	*q*_m_	18	35.31	21.57
		*K*_t_	2.43	1.50	2.10
		*R* ^2^	0.93	0.98	0.981
		MPSD	74.37	55.36	33.24
		NSD	57.61	42.87	25.75
		ARE	47.39	26.18	20.44
Dubinin–Radushkevich	ln *q*_e_ = ln *q*_m_ – βε2	*q*_m_	32.68	94.76	46.10
		β	0.003	0.007	0.004
		ε	13.13	8.22	11.18
		*R* ^2^	0.94	0.99	0.96
		MPSD	32.38	11.24	19.75
		NSD	25.08	9.73	17.10
		ARE	17.77	5.82	11.00
pseudo-first order	ln(*q*_t_ – *q*_e_) = ln *q*_e_ – *k*_1_ *t*	*q*_e_	0.0097	0.0015	0.0105
	$\mathrm{ARE}=\frac{100}{N}\sum_{i=1}^{N}\frac{q_{e}^{\exp}-q_{e}^{\mathrm{cal}}}{q_{e}^{\exp}}$	*k*_1p_	–0.03	–0.06	–0.03
		*R* ^2^	0.09	0.18	0.01
		MPSD	97.24	124.07	37.63
		NSD	86.97	110.97	33.66
		ARE	65.12	110.50	9.68
pseudo-second order	$\frac{1}{q_{t}}=\frac{1}{k_{2}q_{e}^{2}}+\frac{1}{q_{e}}$	*q*_me_	0.41	0.41	0.43
	$\mathrm{MPSD}=100\sqrt{\frac{1}{N-p}}\sum_{i=1}^{N}\left(\frac{q_{ei}^{\exp}-q_{ei}^{\mathrm{cal}}}{q_{ei}^{\exp}}\right)$	*k*_2p_	21.70	5.17	4.77
		*R* ^2^	1	0.99	0.99
		MPSD	0.92	0.87	1.05
		NSD	0.83	0.78	0.94
		ARE	0.05	0.03	0.01
intraparticle diffusion	$q_{t}=k_{p}\sqrt{t}+C$	*k*_p_	0.169	0,174	0.175
	$\mathrm{NSD}=100\sqrt{\frac{1}{N-1}}\sum_{i=1}^{N}\left[\frac{q_{t}^{\exp}-q_{t}^{\mathrm{cal}}}{q_{t}^{\exp}}\right]$	*R* ^2^	1	1	1
		MPSD	45.55	47.31	45.74
		NSD	40.74	42.31	40.91
		ARE	28.90	30.14	29.05
Elovich	*q*_e_ = β ln (αβ) + β ln *t*	β	0.11	0.10	0.13
		α	30.09	33.87	22.14
		*R* ^2^	0.72	0.68	0.72
		MPSD	15.54	26.97	28.07
		NSD	12.69	22.02	25.10
		ARE	4.44	17.54	16.77

### 2.4 Recovery of the Adsorbent

In order to evaluate the reusability of date seed-based adsorbent, four different regeneration methods, given in Table 3, were applied. In each method, 5.0 g of adsorbent was contacted with 250 mL of Rhodamine B solution (4.8 mg L^–1^, pH 3.0) at 303.0 K and 60 rpm for 30.0 min. After adsorption, the regeneration procedure specific to each method was applied, and the residual dye concentration was determined by measuring the absorbance of the supernatant at 553 nm after coarse filtration. All samples were washed five times with deionized water and dried at 333.0 K before reuse. The regeneration process was repeated for four consecutive adsorption–desorption cycles by proportionally reducing the adsorbent mass and solution volume (Cycle 1:5.0 g/250 mL; Cycle 2:4.0 g/200 mL; Cycle 3:3.0 g/150 mL; Cycle 4:2.0 g/100 mL). In order to evaluate the reusability of date seed-based adsorbent, four different regeneration methods were applied.

**Table 3:** Applied Regeneration Methods for Date Seed Adsorbent

**method**	**chemical treatment**	**thermal/microwave treatment**	**conditions**
1. NaOH + thermal (Water bath)^a^	0.01 M NaOH (until neutral pH)	water bath at 60 °C, 60 rpm, 30 min	pH adjusted after adsorption; samples washed (×5) and dried at 60 °C
2. Ethanol + thermal (Water bath)^a^	50 mL ethanol (100%), stirred at 300 rpm, 1 h	water bath at 60 °C, 60 rpm, 30 min	samples washed (×5) and dried at 60 °C
3. NaOH + microwave^a^	50 mL 0.01 M NaOH, stirred 45 min	microwave at 800 W, 2.45 GHz, 5 min	samples washed (×5) and dried at 60 °C
4. NaOH + thermal (Oven)^a^	50 mL 0.01 M NaOH, stirred 45 min	oven at 120 °C, 30 min	samples washed (×5) and dried at 60 °C

a All regeneration experiments were repeated for four adsorption–desorption cycles with gradually reduced adsorbent mass and solution volume (Cycle 1:5 g/250 mL; Cycle 2:4 g/200 mL; Cycle 3:3 g/150 mL; Cycle 4:2 g/100 mL).

The regeneration efficiency (RE, %) was calculated based on the removal efficiency of Rhodamine B in each cycle using the following equation:

$$\mathrm{RE}(\%)=\frac{R_{n}}{R_{1}}\times 100$$ 3

where *R*_1_ and *R*_n_ represent the removal efficiencies (%) in the first and nth cycles, respectively.

## 3 Results and Discussion

### 3.1 Physical Properties, Characterization, and Morphology of Date Seeds

Date seed, a lignocellulosic agricultural waste, is primarily composed of carbohydrates (55–65%), including cellulose, hemicellulose, and lignin, along with crude fiber (10–20%), oil (7–10%), protein (5–7%), moisture (5–10%), and ash (1–2%).^32^ In addition, elemental analysis indicates that date seed is predominantly carbon-rich, containing approximately 85% carbon, with smaller amounts of oxygen, nitrogen, and hydrogen.^32,39^

To better evaluate the performance of the proposed adsorbent, a comparative analysis with previously reported low-cost biosorbents was conducted (Table 4). As shown in Table 4, most Rhodamine B adsorption studies have been performed under mild conditions (25–30 °C), with adsorption capacities generally ranging between 38 and 58 mg g^–1^. The raw date seed used in this study exhibits comparable adsorption performance under similar conditions without any chemical modification. Importantly, unlike most reported biosorbents, which primarily focus on adsorption capacity, the present study provides a systematic evaluation of regeneration and reuse performance. This highlights the practical applicability and sustainability of raw date seed as an effective biosorbent for wastewater treatment.

**Table 4:** Comparison of Rhodamine B Adsorption Performance of Selected Low-Cost Biosorbents under Mild Conditions (25–30 °C)

**adsorbent**	**adsorption capacity** (mg/g)	**pH**	**temperature** (°C)	**regeneration**	**remarks**	**reference**
banana peel powder	38.5	6	25–30	no	raw biomass	^20^
banana peel–based adsorbent	55.3	5–6	30	yes	modified/low-cost biomass	^59^
rice husk	42.1	6	25–30	no	untreated biomass	^22^
rice husk–NaOH treated	1.66	2–11	25–30	no	chemically activated	^60^
coconut shell	54.8	5	30	limited	lignocellulosic biomass	^38^
water hyacinth	58.1	6	25–30	no	aquatic biomass	^61^
sawdust biochar	43.7	6–7	25–30	limited	thermally treated	^62^
date seed activated carbon		2	35	yes	pyrolysis	^63^
raw date seed	32.7	2–8	30	yes	no modification, reusable	This study

The FTIR spectrum of the date seed demonstrates a predominantly oxygenated, polysaccharide-rich surface chemistry (see Table 5). The broad O–H stretching band centered near 3400 cm^–1^, and the pronounced C–O/C–O–C absorptions around 1030 cm^–1^ indicate abundant hydroxyl and ether functionalities deriving from cellulose and hemicellulose. These polar groups increase the material’s hydrophilicity and provide numerous hydrogen-bonding and coordination sites, which are advantageous for the adsorption of polar and cationic species such as Rhodamine B.

**Table 5:** FTIR Band Assignments for Date Seeds^a^

**wavenumber** **(cm** ^**–1**^ **)**	**functional group**	**likely source (biopolymer)**	**interpretation/relevance for adsorption**
∼3400	O–H stretching (broad)	cellulose, hemicellulose, lignin; adsorbed water	presence of hydrogen-bonding hydroxyl groups; contributes to hydrophilicity and provides sites for H-bonding with polar adsorbates (e.g., dyes)
∼2920 and ∼2850	C–H asymmetric and symmetric stretching	aliphatic chains in lignin and extractives	indicates residual aliphatic content; relatively low intensity suggests surface is not dominated by hydrophobic extractives after washing
∼1730	C=O stretching (carbonyl, unconjugated ester)	hemicellulose (acetyl groups), pectin, wax esters	weak or reduced band implies partial removal/deacetylation of hemicellulose; fewer ester carbonyl sites may change interaction with certain cationic species
∼1600	aromatic C=C stretching/conjugated C=O	lignin aromatic skeleton	presence of aromatic functionalities that can provide π–π interactions with aromatic adsorbates (e.g., some dyes)
∼1420–1370	C–H bending/O–H in-plane bending	cellulose, hemicellulose	confirms polysaccharide backbone; contributes to structural rigidity and polar surface chemistry
∼1240–1220	C–O stretching/aryl–O (ether)	lignin (aryl–O), hemicellulose	ether-type functionalities that can participate in dipole interactions and H-bonding
∼1030	C–O–C and C–O stretching (glycosidic)	cellulose, hemicellulose	strong polysaccharide signal—indicates abundant hydroxylated carbohydrate surface groups favorable for H-bonding and metal/dye coordination
∼890	C–H deformation (β-glycosidic linkages)	cellulose	confirms preserved cellulose structure; suggests mechanical stability of the solid substrate

a Note: The spectrum corresponds to the washed date seed sample after removal of surface impurities by distilled water washing.

Weakening or near-absence of intense carbonyl (≈1730 cm^–1^) and reduced aliphatic C–H band intensities imply that washing has removed low-molecular-weight extractives and some hemicellulosic acetyl groups, yielding a cleaner surface with fewer esterified moieties. The remaining aromatic skeletal band near 1600 cm^–1^ suggests lignin domains persist at the surface and may contribute π–π interactions with aromatic adsorbates, complementing hydrogen-bonding and electrostatic adsorption mechanisms. Collectively, the washed date seed presents a balance of polar (−OH, C–O) sites for hydrogen bonding/coordination and residual aromatic structures for π–π interactions. This surface chemistry is consistent with a material that has improved accessibility of polar binding sites after washing, which would be expected to enhance adsorption capacity for polar organic dyes and potentially facilitate subsequent chemical or thermal activation steps.

The BET surface area of the raw date seed was found to be 0.0814 m^2^/g, with a total pore volume of 0.00067 cm^3^/g and an average pore diameter of 13.29 nm. These results indicate that the material possesses a low surface area and weakly mesoporous structure, consistent with other reports on untreated lignocellulosic biomass.^32,40^ The limited nitrogen adsorption can be attributed to pore blockage by lignin, hemicellulose, and organic residues.^41^ The obtained values are in agreement with those reported for raw date seeds (0.02–3 m^2^/g), whereas activated carbons prepared from date seeds generally exhibit surface areas ranging from 300 to 1700 m^2^/g after chemical activation.^42^

The surface charge behavior of raw date seed was investigated by zeta potential measurements over the pH range of 3.0–8.0 to elucidate the adsorption mechanism of RhB (Figure 1). The zeta potential became increasingly negative with rising pH, decreasing from −11.1 mV at pH 3.0 to −35.2 mV at pH 8, indicating progressive deprotonation of surface functional groups. RhB removal efficiency was highest under acidic conditions (pH 3.0–5.0), reaching 94.4–96.3%, and decreased slightly to 88.6% at pH 8.0. This trend is primarily attributed to electrostatic attraction between the negatively charged adsorbent surface and cationic RhB species. Above pH 6.0, partial transformation of RhB to neutral or zwitterionic forms weakens electrostatic interactions, leading to a moderate decline in adsorption efficiency. The combined zeta potential and adsorption results confirm that electrostatic interactions play a dominant role in RhB adsorption onto raw date seed, with an optimal pH range of 3.0–5.0.

**Figure 1:**
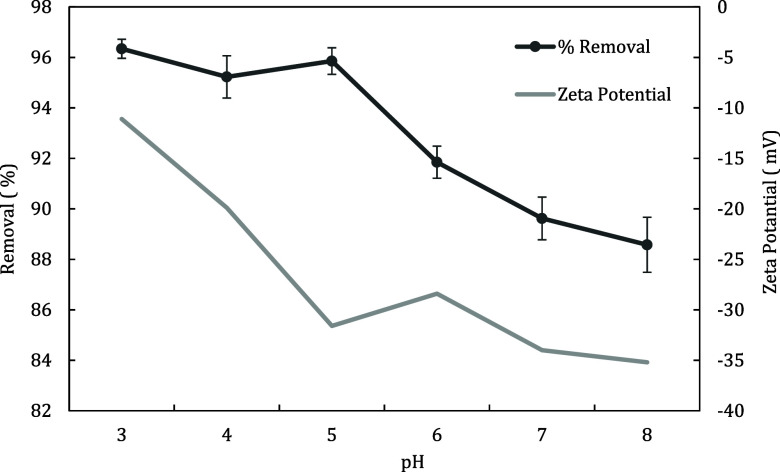
Effect of pH on zeta potential and RhB removal.

SEM images of the raw date seed (Figure 2) show that the natural structure of the adsorbent is well preserved and exhibits a rough and irregular surface morphology. This surface texture indicates the presence of naturally available active sites, which are favorable for adsorption. The heterogeneous structure with microscale voids provides multiple contact points for adsorbate molecules, facilitating surface-controlled adsorption.

**Figure 2:**
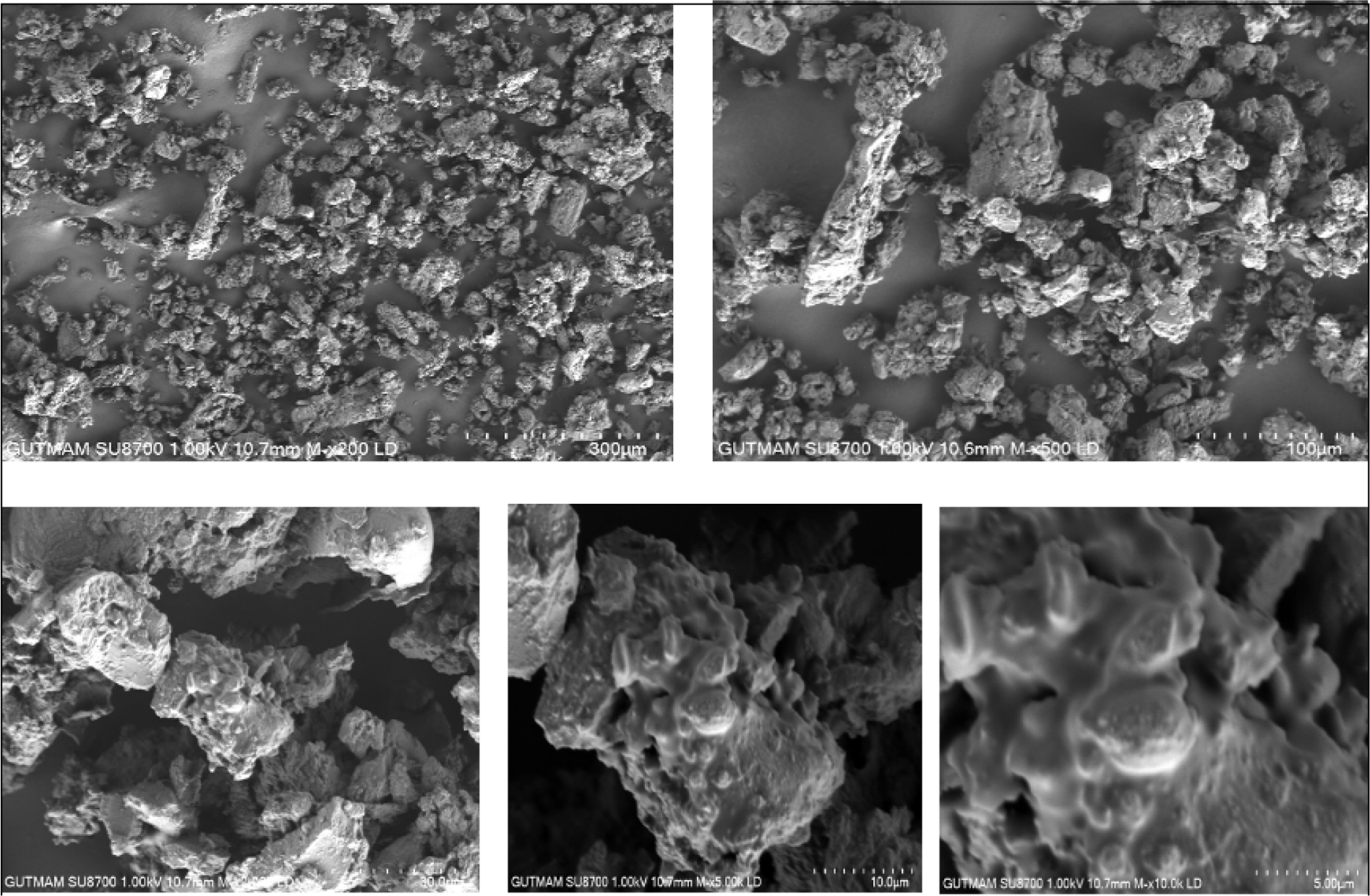
SEM images of the raw date seed at different magnifications (300–5 μm).

### 3.2 Optimization of the Adsorption Parameter

The optimization study was carried out through successive experimental sets to identify the key factors influencing the adsorption performance. In the first step, the effect of pH was examined, and the optimum value was determined as pH 3. Subsequently, keeping the pH constant at 3.0, different amounts of date seed were tested, and 1.0 g was found to be the most effective dosage. In the third step, the contact time was optimized, with maximum adsorption efficiency achieved at 30 min. Finally, the influence of temperature was evaluated, and 303.0 K was identified as the most favorable temperature for RhB adsorption. The optimum results are given in Table 6 and Figure 3.

**Figure 3:**
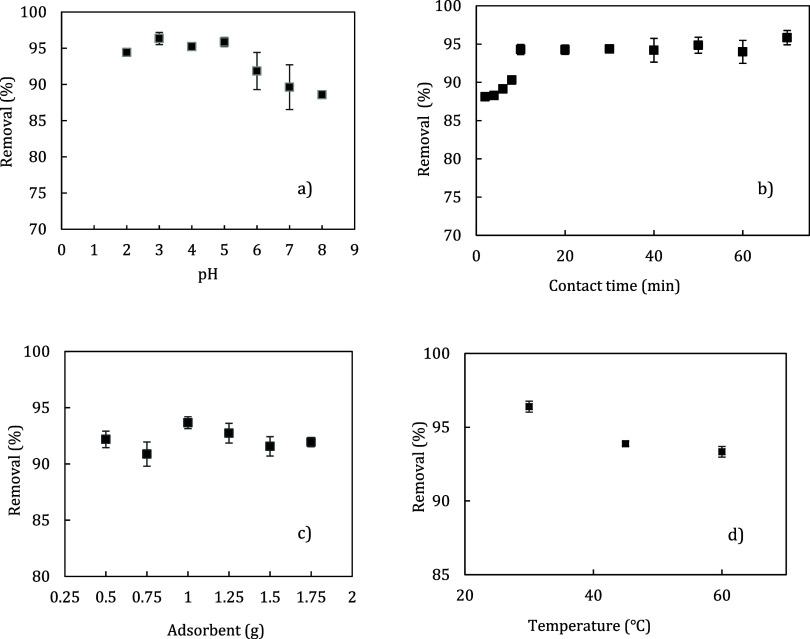
Effect of pH (a), contact time (b), adsorbent dosage (c), temperature (d) on dye removal with date seed.

**Table 6:** Adsorption Experiments Used To Determine the Optimum Operational Conditions

**experiment set**	**fixed conditions**	**tested parameter**	**range of tested values**	**optimum**
set 1		pH	2–8	3
set 2	pH = 3	adsorbent dosage	0.5–1.75 g	1 g
set 3	pH = 3; adsorbent dosage = 1 g	contact time	2–70 min	30 min
set 4	pH = 3; adsorbent = 1 g; time = 30 min	temperature	30–60 °C	30 °C

#### 3.2.1 Effect of pH on the Removal Efficiency of Rhodamine B

The maximum removal efficiency of Rhodamine B (RhB) was observed at pH 3.0, consistent with the molecular speciation behavior of the dye. RhB exists in three structural forms —cationic, lactonic, and zwitterionic—depending on the solution chemistry. In aqueous media, the cationic form dominates under acidic conditions, whereas the zwitterionic species becomes predominant at alkaline pH, and the lactonic form appears only in apolar or polar aprotic environments.^43^ With a p*K*_a_ of 3.7, RhB progressively converts into its zwitterionic form above this pH, which possesses a larger molecular size due to intramolecular electrostatic interactions between the carboxyl and xanthene group.^3,19^ This transformation reduces the effective availability of adsorption sites, leading to a decline in adsorption efficiency at higher pH.

As illustrated in Figure 1, the removal efficiency and zeta potential exhibit consistent trends with pH. The adsorbent surface becomes increasingly negatively charged from pH 3.0 to 8.0 (−11.1 to −35.2 mV), enhancing electrostatic attraction only when RhB remains in its cationic state. The higher adsorption observed below the pHpzc can be primarily attributed to electrostatic attraction between the positively charged adsorbent surface and the negatively charged adsorbate species. However, this mechanism alone does not fully explain the adsorption behavior. At lower pH values, protonation of surface functional groups enhances not only electrostatic attraction but also facilitates additional interactions such as hydrogen bonding and surface complexation. Furthermore, the speciation of the adsorbate under acidic conditions may favor forms that interact more strongly with the adsorbent surface. The highest adsorption efficiency (96% ± 0.38) occurs at pH 3.0, where strong electrostatic attraction is maintained. Above pH 5, although the adsorbent retains a highly negative surface charge, RhB transitions toward its zwitterionic form, resulting in reduced electrostatic affinity and thus lower removal efficiency (88% ± 1.09 at pH 8.0). Conversely, the slightly lower removal at pH 2.0 is attributed to competition between excess H^+^ ions and RhB molecules, which limits dye uptake. Overall, the combined effects of dye speciation and surface charge behavior confirm that pH 3.0 is the optimum condition for RhB adsorption (Figure 3a).

#### 3.2.2 Effect of Contact Time on the Removal Efficiency of Rhodamine B

The influence of contact time on the adsorption of Rhodamine B (RhB) onto raw date seed is illustrated in Figure 3b. A rapid increase in removal efficiency was observed during the first 10 min, which can be attributed to the abundance of accessible active sites and the strong driving force between the dye molecules and the adsorbent surface. As adsorption progressed, the available sites gradually became occupied, resulting in a pronounced decrease in the adsorption rate. Equilibrium was attained at 30 min, beyond which no significant improvement in removal efficiency was observed, indicating the establishment of a dynamic adsorption–desorption equilibrium and reflecting the maximum uptake capacity under the tested conditions.^44^

This adsorption pattern aligns closely with previously reported kinetic behaviors for RhB on low-cost biosorbents^45^ demonstrated that acid-activated mango leaf powder exhibited a similar fast initial phase, followed by a deceleration attributed to repulsive interactions among RhB molecules occupying adjacent surface sites. Supporting evidence from^46^ further indicated that the progressive saturation of active sites on kaolinite decreased the overall adsorption rate as the equilibrium approached. Additional studies on comparable materials reported analogous kinetic trends, with adsorption occurring rapidly at early stages and then diminishing as the system stabilized.^47,48^ Moreover,^49^ emphasized that adequate contact time is essential for effective surface interaction, reinforcing the 30 min equilibrium time identified in this study.

Collectively, these findings confirm that raw date seed exhibits a high affinity for RhB, achieving near-complete removal within a relatively short time frame. The observed kinetic profile, characterized by fast initial uptake followed by gradual site saturation, is fully consistent with established adsorption mechanisms for cationic dyes on lignocellulosic substrates and comparable sorbents reported in the literature.

#### 3.2.3 Effect of Adsorbent Dosage on the Removal Efficiency of Rhodamine B

The effect of adsorbent dosage on the removal performance of Rhodamine B (RhB) was investigated over a range of 0.5–1.75 g, with increments of 0.5 g, as shown in Figure 3c. The removal efficiency increased progressively with the addition of adsorbent up to 1.0 g, where the maximum removal was obtained. This enhancement is primarily attributed to the greater number of available binding sites and the increased effective surface area resulting from the higher solid content in the system, which collectively facilitate more efficient dye–adsorbent interactions.

Beyond 1.0 g, however, the removal efficiency exhibited a slight decline. This decrease is typically associated with particle aggregation or site overlapping at higher dosages, which reduces the accessibility of active adsorption sites even though more adsorbent is present. Excessive adsorbent loading may also induce shielding effects, wherein particles interfere with each other’s adsorption capacity, leading to reduced mass-transfer efficiency. Similar dosage-dependent behavior has been widely reported for RhB and other cationic dyes adsorbed onto lignocellulosic and biobased adsorbents, where an optimum dosage exists, and additional adsorbent does not translate into improved performance.^46–48^ Overall, the obtained results indicate that 1.0 g represents the optimum adsorbent dosage for RhB removal under the studied conditions, providing a balance between sufficient site availability and minimal aggregation-induced reductions in adsorption capacity.

#### 3.2.4 Effect of Temperature on the Removal Efficiency of Rhodamine B

Temperature plays a decisive role in determining the adsorption rate, removal efficiency, and the overall distribution of dye molecules during the adsorption process. In the present study, Rhodamine B (RhB) adsorption was evaluated at 303, 318, and 333 K to investigate how thermal variations influence removal performance. The results clearly demonstrated that increasing temperature negatively affected the adsorption of RhB. As shown in Figure 3d, the adsorption capacity decreased steadily as temperature increased, with the maximum capacity declining from 31.2 ± 0.38 to 30.8 ± 0.36 mg g^–1^ between 303 and 333 K. This reduction indicates that higher temperatures weaken the interactions between RhB molecules and the adsorbent surface, causing diminished retention of dye molecules. RhB molecules more readily overcome the activation barrier and desorb from the adsorbent surface, resulting in a lower removal efficiency. Additionally, higher temperatures under acidic conditions may further suppress the overall reaction rate.^3^ However, this limitation can be partially compensated for by prolonging the contact time. Overall, the decline in adsorption performance with temperature confirms that the process is exothermic, in agreement with previously reported studies on the adsorption of cationic dyes onto lignocellulosic materials.

### 3.3 Adsorption Isotherms and Kinetic Modeling

In order to elucidate the adsorption mechanism of Rhodamine B (RhB) onto date seed, equilibrium and kinetic data were evaluated using widely accepted adsorption models. The isotherm models—including Langmuir, Freundlich, Temkin, and Dubinin–Radushkevich (D–R)—were applied to the equilibrium results obtained at different temperatures (Table 2; Figure 4). Kinetic behavior was further interpreted using pseudo-first-order (PFO), pseudo-second-order (PSO), intraparticle diffusion (IPD), and the Elovich models (Table 2; Figure 5).

**Figure 4:**
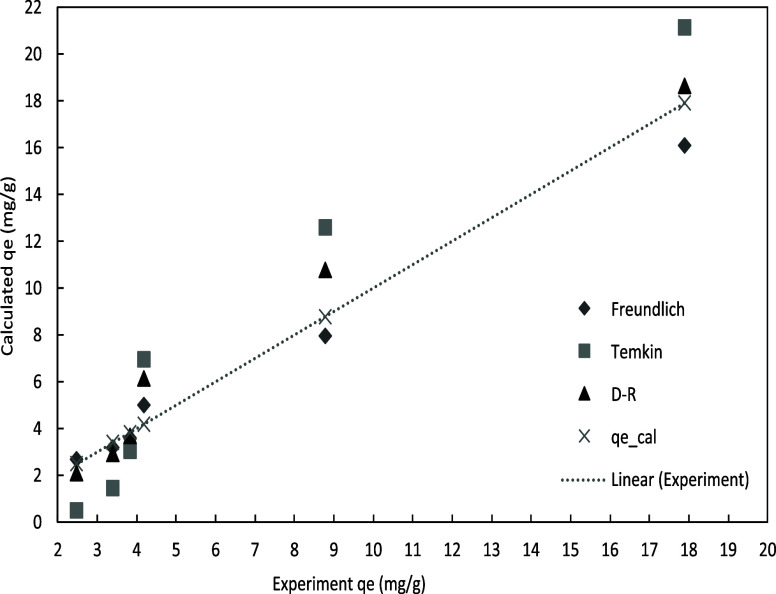
Comparison of experimental and isotherm adsorption capacities.

**Figure 5:**
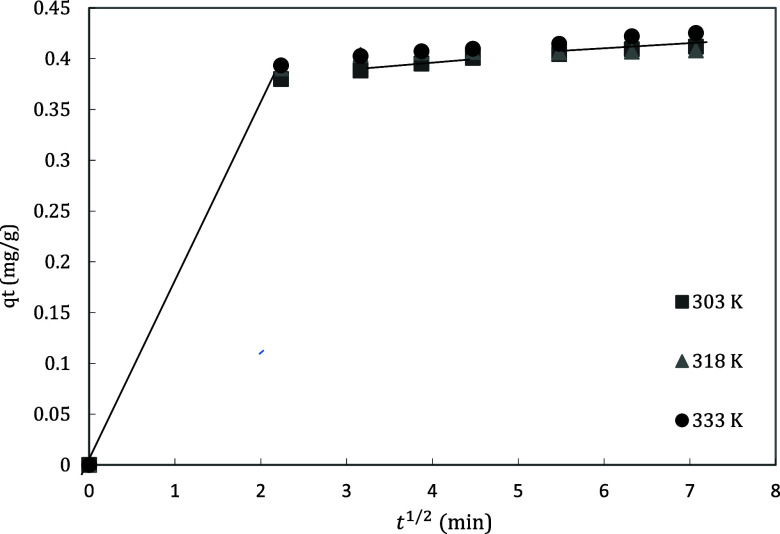
Intraparticle diffusion model on dye removal with Rhodamin B.

#### 3.3.1 Isotherm Analysis

The Freundlich isotherm parameters indicate that Rhodamine B adsorption onto date seed follows a heterogeneous multilayer adsorption mechanism, consistent with previous observations on lignocellulosic adsorbents.^48^ The Freundlich constant (KF) exhibits a temperature-dependent variation, reflecting changes in adsorption affinity toward energetically diverse surface sites. The values of 1/*n* fall within the range of 0 < 1/*n* < 1 at all temperatures, confirming favorable adsorption and the heterogeneous nature of the date seed surface. The relatively high determination coefficients (*R* ^2^ > 0.90) demonstrate that the Freundlich model adequately describes the experimental data. However, the gradual increase in error functions (MPSD, NSD, and ARE) with increasing temperature suggests a decline in model accuracy under thermodynamically less favorable conditions, indicating that adsorption is more efficient at lower temperatures and follows an exothermic trend. Overall, the Freundlich results suggest that the adsorption of RhB onto date seed is heterogeneous, multilayer, and more favorable at lower temperatures, consistent with an exothermic adsorption process.^47^

The Langmuir isotherm parameters demonstrate that Rhodamine B adsorption onto date seed occurs predominantly through monolayer coverage on energetically uniform active sites, consistent with typical cationic dye adsorption behavior on biochar-like materials.^46^ The decrease in Langmuir constants (KL) and maximum monolayer capacity (qm) with increasing temperature indicates a progressive reduction in surface affinity, supporting an exothermic adsorption mechanism. This temperature-dependent decline mirrors previous reports on agricultural waste–derived adsorbents, where higher temperatures disrupt monolayer formation.^47^ The determination coefficients (*R* ^2^ > 0.93 across all temperatures) confirm that the Langmuir model provides a good fit to the experimental isotherm data. However, increasing model error values at elevated temperatures suggest that the assumption of surface homogeneity becomes less valid as thermal agitation weakens the dye–adsorbent interactions. To sum up, the Langmuir isotherm indicates that RhB adsorption onto date seed is primarily monolayer, surface-specific, and strongly favored at lower temperatures, fully consistent with an exothermic adsorption process.

The Temkin isotherm parameters suggest that RhB adsorption onto date seed is governed by temperature-dependent interactions in which the heat of adsorption decreases linearly with increasing surface coverage. This trend is common in dye adsorption onto heterogeneous biobased materials.^44^ The Temkin isotherm parameters reveal that RhB adsorption onto date seed is influenced by adsorbate–adsorbent interactions in which the heat of adsorption decreases linearly with increasing surface coverage. The variation of the Temkin constant with temperature indicates progressive weakening of binding interactions under higher thermal conditions. The high *R* ^2^ values obtained for the Temkin model demonstrate its suitability in describing the energetic heterogeneity of the adsorption system. However, increasing deviations at higher temperatures suggest that interaction-driven adsorption becomes less dominant as the process becomes thermodynamically unfavorable. These results further support an exothermic adsorption mechanism involving moderate binding energies.

The Dubinin–Radushkevich isotherm parameters provide complementary insight into the adsorption mechanism by highlighting the importance of pore-filling processes. The mean adsorption energy values (*E* = 13–8 kJ mol^–1^) suggest that RhB adsorption onto date seed occurs through a mixed mechanism involving ion-exchange interactions, in agreement with findings from similar porous biosorbents.^3,44^ These intermediate energy values imply that both micropore diffusion and surface-level interactions play a role in the adsorption process. The determination coefficients (*R* ^2^ ≈ 0.91–0.93) indicate moderate conformity compared with the Langmuir and Temkin models, suggesting that the D–R model captures pore-related adsorption features but does not fully account for surface heterogeneity. Increasing error metrics at higher temperatures further confirm the reduced significance of pore-filling mechanisms as thermal motion inhibits dye penetration into finer pores.

Overall, a combined evaluation of the isotherm models indicates that Rhodamine B adsorption onto raw date seed proceeds on heterogeneous surfaces via a multilayer mechanism predominantly governed by electrostatic interactions. Among the applied models, the Dubinin–Radushkevich isotherm exhibited the best performance in terms of both statistical conformity and mechanistic relevance, highlighting the contribution of pore-related and energy-dependent adsorption processes. These results clearly demonstrate that raw date seed can act as an efficient biosorbent despite its low BET surface area, emphasizing that adsorption efficiency is controlled not solely by surface area but is strongly influenced by surface chemistry and charge-related properties.

In this study, the experimental adsorption capacities (*q*_e_^exp^) of Rhodamine B onto date seed were compared with the capacities predicted by the Freundlich, Temkin, and Dubinin–Radushkevich (D–R) isotherm models (*q*_e_^cal^). In Figure 4, *q*_e_^cal^ values are plotted against *q*_e_^exp^, where the dashed line represents ideal agreement (*q*_e_^cal^ = *q*_e_^exp^). The proximity of the data points to this line reflects the ability of each isotherm model to describe the experimental data. The Freundlich isotherm systematically underestimated the experimental adsorption capacities, particularly at medium and high *q*_e_ values, indicating that although it effectively describes surface heterogeneity, its quantitative prediction of absolute adsorption capacity is limited. The Temkin model showed significant deviations at low adsorption capacities and tended to overestimate *q*_e_ at higher values, suggesting that its assumption of a linear decrease in adsorption energy with surface coverage is not valid over the entire concentration range. In contrast, the Dubinin–Radushkevich (D–R) model exhibited a closer agreement with the experimental data, although slight overestimations were observed at high *q*_e_ values. Overall, none of the applied isotherm models accurately predicted adsorption capacities across all concentrations; however, the Freundlich and D–R models more consistently captured the experimental trends, confirming that Rhodamine B adsorption on date seed occurs on heterogeneous surfaces through multiple interaction mechanisms that cannot be fully described by a single isotherm model.

#### 3.3.2 Kinetic Modeling

Kinetic modeling provided further insights into the adsorption mechanism. The PFO model showed poor correlation across all experiments, indicating that RhB removal from aqueous solution does not follow first-order adsorption dynamics. In contrast, the PSO model exhibited excellent agreement (*R* ^2^ ≈ 0.999–1.000), with calculated qe values nearly identical to experimental results. This confirms that the rate-limiting step involves chemisorption-like interactions, such as valence force sharing or electron exchange between RhB and the adsorbent surface.^44,45^ Adsorption processes are commonly governed by multiple mechanisms, and kinetic modeling is essential to identify the rate-controlling step and interaction type.^50^

The IPD model indicated a multistep adsorption process. The nonzero intercepts suggest that boundary-layer diffusion governs the early adsorption phase, while intraparticle diffusion contributes significantly during later stages. This pattern is characteristic of dye adsorption onto porous biomass materials.^49^ The Elovich model also showed moderate fitting performance, supporting the presence of heterogeneous active sites and a declining adsorption rate as the surface becomes progressively saturated. Overall, the kinetic findings demonstrate that RhB adsorption onto date seed is controlled by both surface reaction–dominated kinetics and diffusion-related mechanisms, with the PSO model providing the most accurate description. By integrating both isotherm and kinetic results, RhB adsorption onto date seed can be described as an exothermic, monolayer-driven process occurring on heterogeneous surfaces, with adsorption controlled by chemically influenced surface interactions and multistep diffusion mechanisms. The combined model interpretation is consistent with previous findings for biochar and lignocellulosic waste–based adsorbents reported in the literature.^3,48^

#### 3.3.3 Removal Mechanism

An examination of the intraparticle diffusion rate constants obtained at different temperatures clearly indicates that the adsorption-based removal process proceeds through a multistage mechanism. As seen Figure 5, in the first linear region, the relatively high diffusion rates calculated at 303, 318, and 333 K (0.169, 0.174, and 0.175 mg g^–1^ min^–1^, respectively) suggest that, during the initial stage of removal, adsorbate molecules are rapidly transported to the external surface of the adsorbent, with film diffusion and surface adsorption being the dominant mechanisms. Over time, the pronounced decrease in diffusion rates observed in the second linear region (0.009, 0.002, and 0.007 mg g^–1^ min^–1^) indicates that the adsorbate molecules begin to diffuse into the pore structure of the adsorbent, rendering intraparticle diffusion the rate-controlling step. In the final linear stage, the further reduction in diffusion rates (0.005, 0.001, and 0.006 mg g^–1^ min^–1^) reflects that most of the active sites have been occupied and the system approaches equilibrium, during which the removal rate is significantly reduced due to mass transfer limitations. The slight increase in diffusion rates in the initial stage with increasing temperature suggests that the removal process is thermally assisted and that the adsorption may exhibit an endothermic character.

The intraparticle diffusion analyses reveal that film diffusion dominates the initial stage of adsorption, whereas pore diffusion becomes increasingly influential in the subsequent stages. The gradual decrease in diffusion rates over time indicates the progressive occupation of active sites on the adsorbent surface by RhB molecules and the system’s approach toward equilibrium. This multistage behavior suggests that the adsorption process is not limited solely to physical attachment but also involves surface complexation and chemical interactions.

The obtained kinetic and intraparticle diffusion results demonstrate that the removal of Rhodamine B (RhB) by the date seed adsorbent occurs through a multimechanistic adsorption process. The excellent agreement of the pseudo-second-order kinetic model with the experimental data at all temperatures, reflected by high correlation coefficients (*R* ^2^ ≈ 0.99–1.00), indicates that the rate-limiting step of the adsorption process is associated with surface interactions and chemical bonding mechanisms. This finding suggests strong interactions between RhB molecules and the active functional groups present on the adsorbent surface.

The FTIR spectral analysis shows a broad band around 3400 cm^–1^, which is attributed to the presence of hydroxyl (−OH) groups derived from cellulose, hemicellulose, and lignin.^51^ These functional groups significantly contribute to the adsorption process by facilitating hydrogen bond formation with RhB molecules. The bands observed at 2920 and 2850 cm^–1^ correspond to asymmetric and symmetric C–H stretching vibrations associated with lignin and aliphatic structures (see Figure 6). Their relatively low intensities indicate that the surface is not dominated by hydrophobic extractives, suggesting a predominantly polar surface character that is favorable for the adsorption of polar or ionic dye molecules such as RhB. The aromatic C=C stretching vibrations observed around 1600 cm^–1^ confirm the preservation of the lignin structure and the presence of aromatic rings on the adsorbent surface. Considering the aromatic structure of RhB molecules, these regions facilitate π–π interactions, which serve as an important mechanism enhancing the adsorption capacity.^52^ The C–H and O–H bending vibrations in the range of 1420–1370 cm^–1^ further confirm the integrity of the polysaccharide backbone (cellulose and hemicellulose) and support the polar nature of the surface given in Figure 6. Similarly, the bands observed between 1240 and 1220 cm^–1^, attributed to C–O and aryl–O vibrations originating from lignin and hemicellulose ether groups, indicate the presence of functional groups that contribute to RhB adsorption through dipole–dipole interactions and hydrogen bonding. The strong bands around 1030 cm^–1^, corresponding to C–O–C and C–O stretching vibrations, reveal the presence of glycosidic linkages derived from cellulose and hemicellulose and indicate a high abundance of hydroxylated carbohydrate groups on the adsorbent surface. These groups support hydrogen bonding and surface complexation mechanisms with RhB molecules. The band observed near 890 cm^–1^ confirms the preservation of β-glycosidic linkages, thereby validating the structural integrity of the adsorbent.

**Figure 6:**
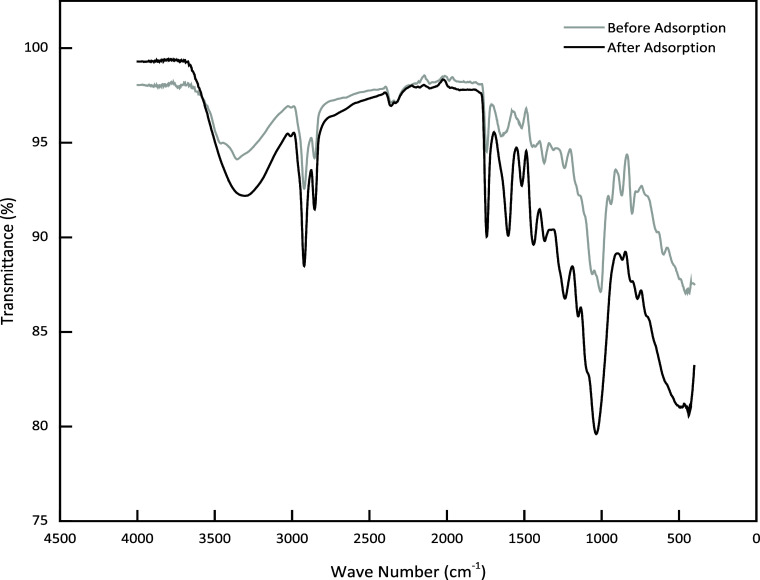
FTIR spectra of raw date seed before and after adsorption.

The obtained findings demonstrate that RhB removal by the date seed adsorbent proceeds through a multimechanistic process involving film diffusion, intraparticle diffusion, and surface interactions acting simultaneously. These results are consistent with the high fitting accuracy of the pseudo-second-order kinetic model and the observed intraparticle diffusion behavior, confirming the effective removal of RhB by date seed adsorbent.^53^ The findings clearly indicate that RhB removal is not limited to physical adsorption alone but occurs through a multimechanistic adsorption process involving hydrogen bonding, π–π interactions, dipole interactions, and surface complexation.

### 3.4 Thermodynamic Interpretation

The influence of temperature on the adsorption behavior was evaluated through thermodynamic parameters, namely Gibbs free energy change (Δ*G*°), standard enthalpy change (Δ*H*°), and standard entropy change (Δ*S*°). These parameters were calculated using eqs 4–8, and the obtained values are summarized in Table 7.^54,55^

**Table 7:** Thermodynamic Analysis of Adsorption

Temperature (K)	Δ*G*° (kJ/mol)	Δ*H*° (kJ/mol)	Δ*S*° (J/mol·K)
298	–1.58	–6.10	–0.70
318	–1.53		
333	–1.57		

The Gibbs free energy change was determined using eq 4:

$$\Delta G^{o}=-RT\;\ln\;K_{d}$$ 4

where *R* is the universal gas constant (8.314 J/mol K), *T* is the absolute temperature (*K*), and *K*_d_ is the distribution coefficient. The relationship between *K*_d_, enthalpy, and entropy is expressed by the van’t Hoff equation (eq 5):

$$\ln\;K_{d}=\frac{\Delta S^{o}}{R}-\frac{\Delta H^{o}}{RT}$$ 5

Additionally, the thermodynamic parameters are related through eq 6:

$$\Delta G^{o}=\Delta H^{o}-T\Delta H^{o}$$ 6

The distribution coefficient *K*_d_ was calculated using eq 7:

$$K_{d}=\frac{C_{\mathrm{Ae}}}{C_{e}}$$ 7

where *C*_e_(mg L^–1^) represents the equilibrium concentration of RhB in solution and *C*_Ae_ denotes the amount of dye adsorbed per unit volume at equilibrium.

The values of Δ*H*° and Δ*S*° were obtained from the slope and intercept of the linear plot of ln *K*_d_ versus 1/*T*, respectively.

The thermodynamic parameters indicate that the adsorption of Rhodamine B onto raw date seed is a spontaneous process at all investigated temperatures, as evidenced by the negative Δ*G*° values. The relatively small magnitude of Δ*G*° suggests that the adsorption is mainly governed by physical interactions rather than chemical bonding. The negative enthalpy change (Δ*H*° = −6.10 kJ/mol) confirms the exothermic nature of the adsorption process, implying that lower temperatures favor dye uptake. Furthermore, the slightly negative entropy change (Δ*S*° = −0.70 J/mol·K) indicates a minor decrease in randomness at the solid–liquid interface during adsorption, which can be attributed to the orderly attachment of Rhodamine B molecules onto the adsorbent surface (Table 7).

Furthermore, the minimum energy required for the interaction between RhB molecules and the active sites of the sorbent, known as the Arrhenius activation energy (*E*_a_), was evaluated. The activation energy for RhB adsorption onto PBC was calculated using the pseudo-second-order rate constants (*k*_2_) obtained at different temperatures, according to the Arrhenius equation (eq 8):^55^

$$\ln\;k_{2}=\ln A+\frac{E_{a}}{RT}$$ 8

where *A* is the Arrhenius pre-exponential factor, *E*_a_ (kJ mol^–1^) is the activation energy, and *k*_2_ represents the pseudo-second-order rate constant. The plot of ln *k*_2_ versus 1/*T* was used to determine *E*_a_ from the slope and *A* from the intercept, providing insight into the nature of the adsorption process. The activation energy is approximately 0.94 kJ mol^–1^. The low activation energy indicates that the adsorption process is dominated by physical interactions rather than chemical bonding.

### 3.5 Reusability and Regeneration Performance of Date Seed

The reusability of the date seed–based adsorbent was systematically evaluated under four different regeneration strategies, and its performance was monitored over four consecutive adsorption–desorption cycles. The regeneration conditions applied in this study are summarized in Table 8. The regeneration efficiency exhibited clear method-dependent differences, directly reflecting the chemical and structural stability of the adsorbent under repeated use. Across all methods, NaOH-based regeneration consistently provided superior desorption performance, whereas ethanol-based treatment yielded noticeably lower efficiencies.

**Table 8:** Comparison of Desorption Efficiencies of Various NaOH- and Ethanol-Based Regeneration Methods Reported in This Study and in the Literature

regeneration method	adsorbent type	dye	desorbent treatment	desorption efficiency (%)	cycles	reference
ethanol + thermal	raw date seed	rhodamine B	100% ethanol (60 min) + 60 °C water bath (45 min)	74–79	4	this study
NaOH + thermal (60 °C)	raw date seed	rhodamine B	0.01 M NaOH (45 min) + 60 °C water bath (45 min)	84–92	4	this study
NaOH + thermal (120 °C)	raw date seed	rhodamine B	0.01 M NaOH (45 min) + 120 °C oven (30 min)	88.1–89.1	4	this study
NaOH + microwave (800 W)	raw date seed	rhodamine B	0.01 M NaOH (45 min) + microwave (800 W, 5 min)	88.3–89.3	4	this study
ethanol + thermal	orange peel	methylene blue	ethanol (1 h) + mild heating	68	2	Nguyen et al., 2019
ethanol	wheat bran	rhodamine B	100% ethanol wash	62	1	Kaur and Sud, 2020
NaOH + microwave (700 W)	orange peel	methylene blue	0.1 M NaOH + microwave (700 W, 3 min)	85	3	Ali et al., 2021
NaOH + thermal (100 °C)	rice husk	rhodamine B	0.1 M NaOH + 100 °C water bath (60 min)	87	3	Zhang et al., 2022
NaOH + thermal (110 °C)	corn cob	rhodamine B	0.1 M NaOH + 110 °C oven (30 min)	90	4	Patel et al., 2018
NaOH + microwave (850 W)	peanut shell	methylene blue	0.05 M NaOH + microwave (850 W, 4 min)	86	2	Chen et al., 2020

In the chemical–thermal route using 0.01 M NaOH followed by treatment in a 333.0 K water bath, the desorption efficiency decreased only slightly from 91.6% in the first cycle to 83.8% in the fourth cycle in Figure 7. This minimal loss indicates that NaOH effectively disrupts electrostatic interactions and weak intermolecular forces between RhB molecules and the adsorbent surface. Increasing pH promotes deprotonation of functional groups, enhancing surface negativity and causing repulsion with cationic RhB, which facilitates desorption. Thermal activation at 333.0 K further enhances desorption by mobilizing dye molecules trapped in shallow pores. This combined action explains the high and stable performance observed throughout the cycles. In contrast, regeneration with ethanol followed by the same thermal treatment displayed the lowest performance among all methods, showing a decrease from 78.8 to 72.9% over four cycles. The evolution of removal efficiencies is shown in Figure 7. Ethanol’s limited polarity and insufficient ability to disrupt ionic interactions significantly weaken its desorption capability. Since RhB forms strong electrostatic and π–π interactions with the date seed surface, a nonalkaline, low-polarity solvent provides inadequate driving force for dye removal. Adsorption mechanisms involving electrostatic interactions, hydrogen bonding, and π–π interactions have been widely reported for biomass-based adsorbents.^56^ This poor performance is consistent with the literature, indicating that ethanol is generally ineffective for regenerating biochar-based adsorbents used for cationic dyes.

**Figure 7:**
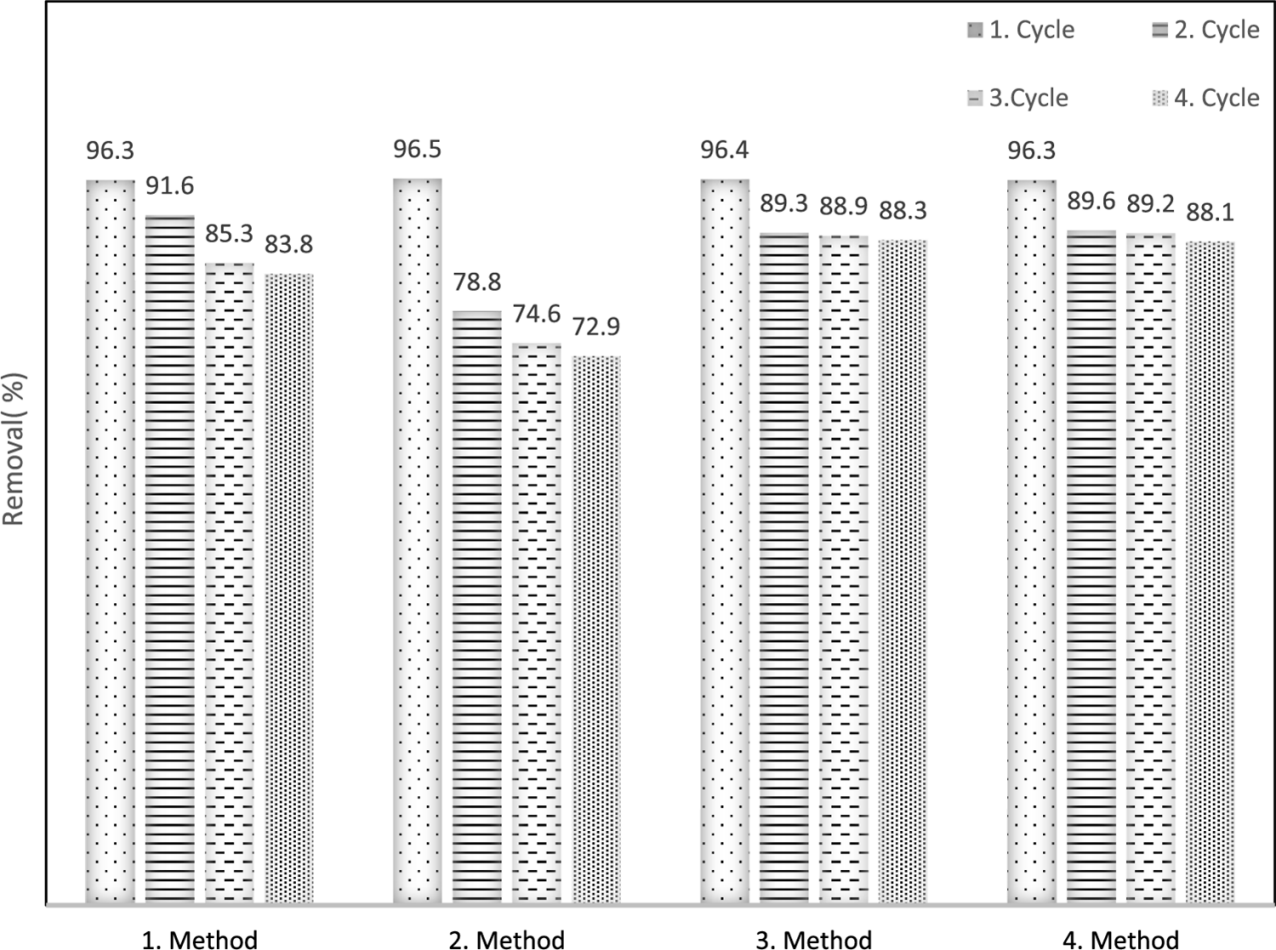
Removal efficiency (%) of Rhodamine B by raw date seed after four successive regeneration cycles using four different regeneration methods.

When microwave irradiation (800 W for 5 min) was combined with NaOH treatment, the regenerated adsorbent exhibited exceptionally stable performance, decreasing only from 89.3 to 88.3% given in Figure 7. Microwave energy creates localized heating hotspots within the carbonaceous matrix, accelerating the penetration of NaOH into internal pores and promoting rapid desorption. The shorter regeneration time and minimal efficiency loss highlight microwave-assisted chemical regeneration as a highly energy-efficient and scalable method. Compared with thermal treatments, microwave treatment avoids structural collapse while still enhancing desorption kinetics, which aligns with reports demonstrating improved regeneration efficiency through targeted microwave heating. The fourth method, which combined NaOH treatment with high-temperature drying at 120 °C, also showed high desorption efficiency (89.1 to 88.1%) in Figure 7. Although effective, this approach requires significantly more energy than microwave heating and may pose challenges in large-scale applications. Nevertheless, the stability of removal efficiency indicates that the date seed adsorbent retains its structural integrity even under harsher thermal conditions.

When the four methods are compared collectively, NaOH-based chemical regeneration clearly outperforms solvent-based and temperature-only strategies. The enhanced performance is attributed to the strong interaction between NaOH and ionizable groups on the adsorbent surface, which promotes desorption through electrostatic repulsion, hydrogen bond disruption, and partial ion exchange. Ethanol-based regeneration, on the other hand, fails to efficiently remove RhB due to its inability to disrupt these interactions. Figures 7, representing removal and desorption efficiency trends across cycles, demonstrate that Methods 1, 3, and 4 yield high and stable performance, whereas Method 2 shows a progressive decline. The date seed–based adsorbent demonstrates excellent chemical stability and can be successfully regenerated and reused through NaOH-assisted processes, particularly when combined with microwave heating. The strong regeneration performance confirms its suitability for repeated application in RhB removal, enhancing its practicality as a low-cost and sustainable adsorbent for wastewater treatment.^57^

#### 3.5.1 Surface Morphology after Regeneration

SEM analyses were conducted to evaluate the surface morphological changes of the date pit–based adsorbent after adsorption and desorption processes applied under four different regeneration methods. Following RhB adsorption, all samples exhibited a generally rough, irregular, and heterogeneous surface morphology, indicating that adsorption occurred not only on the external surface but also within the pore structure. In the first regeneration method, adsorption led to partial pore filling and localized surface compaction, with film-like deposits and blocked micropores becoming more pronounced at higher magnifications. After desorption, the surface morphology partially recovered, suggesting that a significant fraction of the adsorbed RhB molecules was successfully removed and that structural reversibility was moderately preserved.

In contrast, the second regeneration method resulted in more severe morphological alterations. Postadsorption SEM images revealed extensive blockage of micro- and mesopores and a markedly compact surface structure. Although desorption led to partial reopening of the surface, several pores remained obstructed, and localized surface collapses were observed, particularly at higher magnifications. These features indicate strong dye–adsorbent interactions and limited effectiveness of the regeneration process in restoring the original pore structure.^58^

The third regeneration method also induced notable morphological degradation. After adsorption, localized surface compaction, pore mouth blockage, and film-like RhB deposits were evident, confirming intensive surface and intraparticle adsorption. Following desorption, only partial recovery of the surface was achieved, while increased surface roughness, erosion-like features, particle agglomeration, and reduced pore continuity were observed. These irreversible morphological changes suggest a decline in pore accessibility and structural integrity after regeneration. Among all methods, the fourth regeneration method demonstrated the most favorable morphological performance (Figure 8). After adsorption, the adsorbent largely retained its rough and heterogeneous structure, with only partial filling of micro- and mesopores and no significant surface collapse. Desorption effectively reopened most of the pores, and the surface morphology closely resembled that of the fresh adsorbent. Minimal permanent pore blockage was observed at higher magnifications, indicating efficient removal of adsorbed RhB molecules.

**Figure 8:**
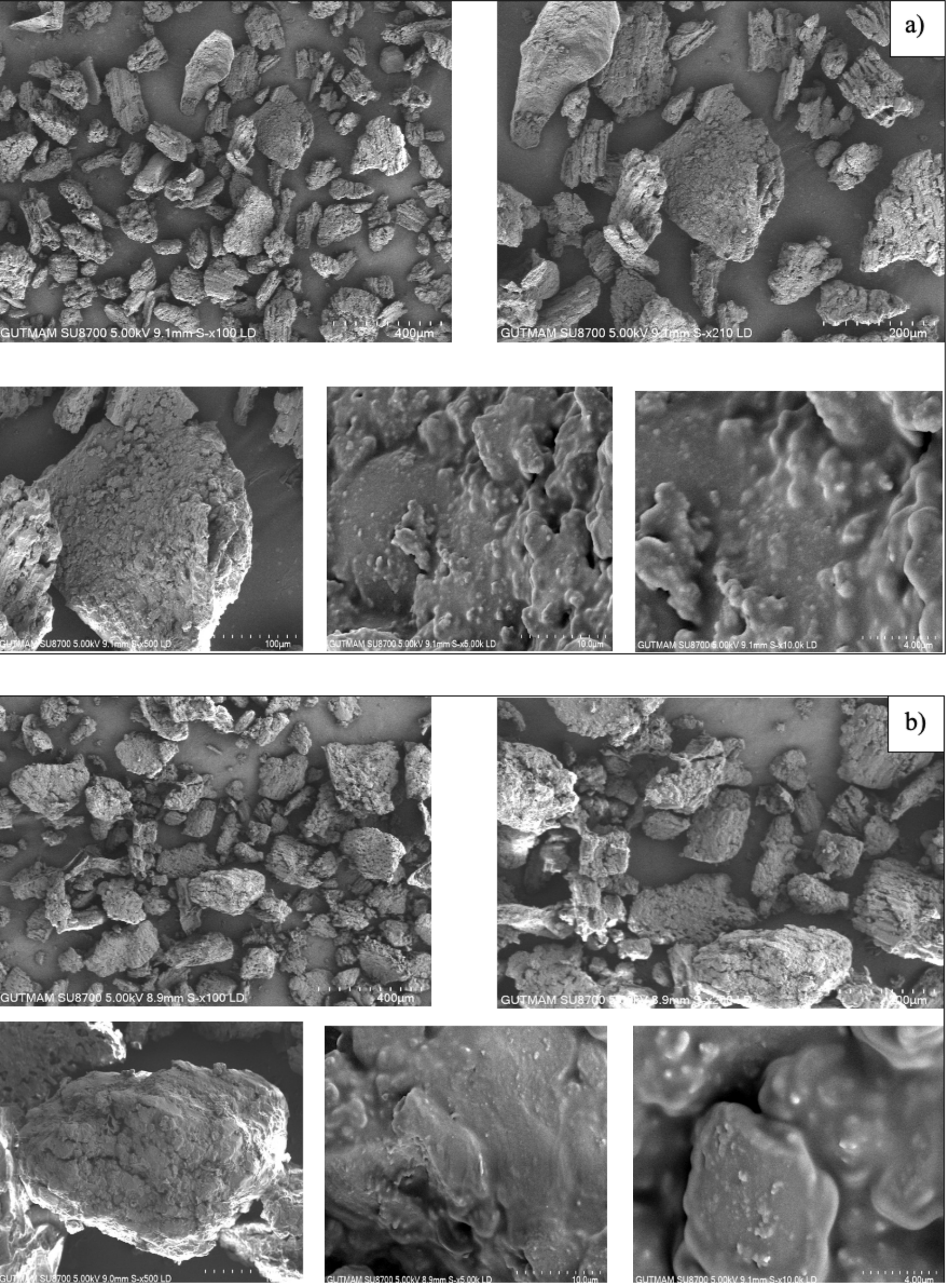
SEM images illustrating the surface morphological changes of the date seed–based adsorbent after (a) adsorption and (b) desorption processes within the fourth regeneration method.

SEM and BET (Table 9) analyses jointly demonstrate that the adsorption and regeneration processes induce significant structural and morphological alterations in the date seed adsorbent. SEM images of the raw material reveal an irregular, fibrous lignocellulosic structure with well-defined micro- and mesopores, consistent with the BET values indicating a surface area of 0.0814 m^2^g^–1^ and a pore diameter of 13.29 nm. After four adsorption–desorption cycles, all regenerated samples exhibit varying degrees of pore blockage, surface smoothing, and partial collapse of structural channels, in agreement with the substantial reduction in surface area across all methods. BET results further support these observations. Samples regenerated using Method 2 and Method 3 did not show BET surface areas. SEM micrographs of these samples typically display dense, compacted surfaces with reduced porosity, indicating that the structural network was altered due to thermal and mechanical stress during regeneration. In contrast, Method 4 exhibited the most effective structural recovery, with a BET surface area of 0.0398 m^2^g^–1^ and a higher pore volume compared to other regenerated samples. SEM images of Method 4 show partially reopened pore channels and fewer collapsed regions, consistent with the observed textural improvement. High-temperature regeneration at 393.0 K appears to effectively desorb strongly retained RhB molecules while preventing excessive structural distortion. In Figure 9, the FTIR spectra of raw date seed before adsorption and after adsorption–desorption in the fourth regeneration cycle show slight shifts and intensity changes in several characteristic bands, suggesting the involvement of surface functional groups in the adsorption process of Rhodamine B. The broad band in the range of 3200–3500 cm^–1^, assigned to O–H stretching vibrations, exhibits a decrease in intensity after adsorption, indicating possible interactions between hydroxyl groups on the adsorbent surface and dye molecules. Minor variations observed at 2920–2850 cm^–1^, corresponding to C–H stretching vibrations, imply weak interactions with the organic framework of the adsorbent. Additionally, changes in the region of 1600–1500 cm^–1^ may be associated with interactions between the aromatic structures of Rhodamine B and the carbonaceous matrix of the raw date seed. The partial restoration of band intensities after desorption suggests that these interactions are largely reversible, supporting the regenerability of the adsorbent without significant alteration of its surface functional groups.

**Table 9:** Comparison of the BET Surface Properties of Raw and Regenerated Date Seed Adsorbent after the 4th Cycle

sample/method	BET surface area (m^2^/g)*A*	total pore volume (cm^3^/g)	average pore diameter (nm)
raw date seed	0.0814	0.000670	13.29
method 1–chemical + thermal (60 °C)	0.0194	0.000426	19.32
method 2–ethanol + thermal (60 °C)	N.A	0.000357	20.26
method 3–chemical + microwave (800 W)	N.A	0.000431	29.65
method 4–chemical + high-temp thermal (120 °C)	0.0398	0.000606	16.98

**Figure 9:**
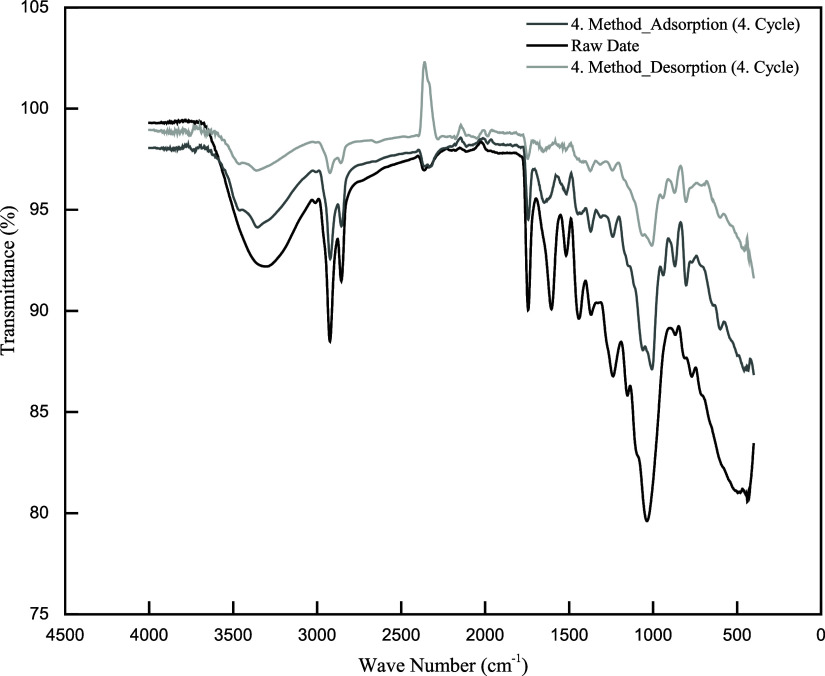
FTIR spectra of raw date seed and 4. regeneration method adsorption–desorption (4. cycle).

Overall, the combined SEM–BET findings reveal that adsorption of RhB leads to pore blockage and reduction of accessible surface area, whereas the choice of regeneration method dictates the extent of structural degradation or recovery. Method 4 offers the best preservation of the original pore architecture, while Methods 2 and 3 show significant damage or pore congestion. These textural changes directly correlate with desorption efficiencies and highlight the importance of selecting regeneration strategies that balance chemical effectiveness with structural stability.

## 4 Conclusions

The present search demonstrated that Rhodamine B (RhB) adsorption onto raw date seed is governed by a multimechanistic process, primarily driven by electrostatic interactions and supported by hydrogen bonding, π–π interactions, and surface complexation. The adsorption behavior was best described by the Freundlich and Dubinin–Radushkevich isotherm models, indicating a heterogeneous and multilayer adsorption process, while kinetic analysis confirmed that the process follows a pseudo-second-order model (*R* ^2^ = 1), suggesting surface-controlled adsorption. The adsorption performance was strongly influenced by solution pH, with optimum removal observed at pH 3–5 due to enhanced electrostatic attraction between the negatively charged adsorbent surface and cationic RhB molecules. Under optimal conditions (pH 3, 30 °C, 30 min, 60 rpm, and 1 g adsorbent), a maximum removal efficiency of 96% was achieved. Thermodynamic analysis indicated that the adsorption process is exothermic and more favorable at lower temperatures.

Importantly, the regeneration results showed that adsorbent performance is highly dependent on the regeneration strategy, with the NaOH-assisted thermal regeneration method at 120 °C (fourth method) providing the highest adsorption recovery and structural stability. The RhB removal efficiencies obtained using this method were 96.3% in the first cycle and 88.1% after the fourth cycle, demonstrating its effectiveness for repeated use. This study provides a comparative evaluation of regeneration methods for raw date seed–based adsorbents, demonstrating that surface chemistry plays a more decisive role than surface area in RhB removal. The findings confirm that raw date seed is a low-cost, sustainable, and efficient biosorbent with strong potential for practical wastewater treatment applications. Future studies should focus on evaluating the performance of regenerated adsorbents over extended cycles, analyzing their applicability to different classes of pollutants, and assessing their efficiency under real wastewater conditions.

## Data Availability

The data that support the findings of this study are available from the corresponding author upon reasonable request.
